# A retrospective survey of death care practices and procedures in handling suspected and confirmed COVID-19 deceased bodies in the mortuary of a resource-poor tertiary healthcare facility in Uyo, South-South Nigeria

**DOI:** 10.11604/pamj.2022.41.176.32111

**Published:** 2022-03-04

**Authors:** Uchechukwu Brian Eziagu, Ikwo Kudamnya, Asukwo Etim Onukak, Chinedu Onwuka Ndukwe

**Affiliations:** 1 Department of Histopathology, University of Uyo Teaching Hospital, Uyo, Akwa Ibom State, Nigeria,; 2 Department of Internal Medicine, University of Uyo Teaching Hospital, Uyo, Akwa Ibom State, Nigeria,; 3 Department of Anatomic Pathology and Forensic Medicine, Nnamdi Azikiwe University, Awka/Nnewi, Anambra State, Nigeria

**Keywords:** COVID-19, SARS-CoV-2, death care practices and procedure, deceased bodies, embalmment, mortuary, Nigeria

## Abstract

**Introduction:**

the death care of deceased bodies of confirmed COVID-19 cases is a critical component of holistic healthcare provision for COVID-19 cases particularly from the public health perspective of infection prevention/control. Regrettably, there is a dearth of research-based information on the management (death care) of COVID-19 deceased bodies. Thus, we aimed to determine the preservation/storage death care practices and procedures in accordance with infection prevention/control measures used for deceased bodies of suspected/confirmed COVID-19 cases deposited in our mortuary and explore their antemortem age/sex distribution.

**Methods:**

a retrospective hospital-based cross-sectional survey done at the mortuary unit of Department of Histopathology, University of Uyo Teaching Hospital (UUTH), Uyo, Akwa Ibom State (covering January 2020 to September 2021). Our study participants were all deceased bodies of suspected/confirmed COVID-19 cases deposited in/cared for at UUTH mortuary within this period.

**Results:**

we found 28 suspected/confirmed COVID-19 deceased bodies, of which 51.86% were within the 6^th^ and 7^t h^decade of life and 71.43% were males (M/F = 2.5/1). They constituted 5.93% of the deceased bodies deposited in UUTH mortuary within this period. All (100%) were embalmed, and 75% were embalmed by immersion. The peak periods of their deposition were within the first halves of the two years affected by the COVID-19 pandemic.

**Conclusion:**

we provided death care (mainly through embalmment) for deceased bodies of suspected/confirmed COVID-19 cases in our resource poor setting using locally derived innovative means while still adhering to infection prevention/control measures to protect our death care workers in the mortuary.

## Introduction

COVID-19 is a primary infectious lung disease, caused by severe acute respiratory syndrome coronavirus 2 (SARS-CoV-2). SARS-CoV-2 is an ribonucleic acid (RNA) virus of the enveloped ß-coronavirus family. COVID-19 presentation range from asymptomatic to critical respiratory/multisystemic syndrome (leading to death) [1]. SARS-CoV-2 is a Hazard Group 3 (HG3) or Risk Group 3 (RG3) pathogen/biological agent (just like MERS coronavirus, Hepatitis B, C, D and E viruses, HIV 1 and 2, Polio virus, Dengue virus, Rabies virus, and *Mycobacterium tuberculosis*). It is transmitted from human to human through close/direct contact, respiratory droplets/aerosols, contaminated materials/surfaces (fomites) and, possibly faeces [2-7]. Tragically, since the beginning of this COVID-19 pandemic, over 4.6 million persons have died out of about 227 million confirmed cases worldwide [1,8]. Presently, as at September 2021, United States of America (USA) and India are the two worst hit countries with the highest number of deaths from COVID-19, having confirmed deaths/confirmed cases values as 663,141/41,395,425 and 444,248/33,381,728 respectively [8]. Presently too, in China, the pandemic´s ground zero, the confirmed deaths/confirmed cases value is 5,688/123,904 [8]. Similarly, in Nigeria, the confirmed deaths/confirmed cases value is 2,654/201,630 [9]. Notably in Uyo, the confirmed deaths/confirmed cases value is 42/4,284 [9]. In Nigerian, COVID-19 affected mainly males and individuals with median age of 43 years (southwestern Nigeria) and mean age of 41.5 years (south-south Nigeria) [10,11]. Similarly, data from around the world show that most of the deaths occurred in males and in individuals aged 65 years and above [12].

While ample research-based information are available for management (treatment and infection prevention/control) of COVID-19 patients (until their death), there are few research-based information on the management (death care) of deceased bodies of COVID-19 patients [13,14]. Notably, death care of the deceased bodies of COVID-19 patients is a critical component of the holistic healthcare of COVID-19 cases particularly from public health perspective of infection prevention/control [2]. Presently, death care for COVID-19 deceased bodies is based on numerous interim guidelines provided by the World Health Organization (WHO), International Committee of the Red Cross (ICRC), Public Health England, Royal College of Pathologist (RCPath), Nigeria Center for Disease Control (NCDC), etc. [2-5,7,13-29]. Death care providers/workers (professionals and non-professionals) are all individuals directly handling these deceased bodies [6,27,29]. They include Anatomical Pathologists, Morticians, funeral homes and cemeteries staff, etc. [5,6,16,29,30].

The death care process (practices and procedures) in handling these COVID-19 deceased bodies differs from non-COVID-19 deceased bodies because of application of infection prevention/control measures, although the infectivity potential of COVID-19 deceased body remains controversial [2,4,6,7,15,22,25,27-29]. These guidelines outline pertinent infection prevention/control measures for death care workers as well as technical recommendations for the death care process. Infection prevention/control measures include proper hand hygiene, use of personal protective equipment (PPE), body bags, and plastic/cloth sheeting to cover deceased bodies. These PPE include aprons/long-sleeved gowns, eye googles/face shields, face masks (FFP2, FFP3, and N95 respirators), surgical gloves, and closed toe shoes/rubber boots [2,4-6,22, 27-29]. The stages of the death care process, as outlined in the guidelines, include: transportation of the deceased from death site, reception at mortuary/funeral home, preservation/storage at the mortuary/funeral home, release from the mortuary, burial/cremation, autopsy/post-mortem examination, etc.[4-6,22,27-29]. This study focuses on the death care process (practices and procedure) stages of reception and preservation/storage of COVID-19 deceased bodies at the mortuary in accordance with infection prevention/control measures. Preservation of deceased bodies entails processes/procedures employed to prevent decomposition/decay until the time of burial/cremation, either by use of chemicals (embalmment) or physical agents such as cold (body freezer) [31-33]. Embalmment is performed by using embalming fluids through various methods, namely: immersion, arterial injection, cavity injection and surface injection [31-33]. Notably, formaldehyde-based embalming fluid types are the most excellent embalming fluids for deceased body preservation and minimization of transmission of infection from deceased body [31,32]. In this study, we aimed to determine the preservation/storage methods and infection prevention/control measures deployed in the death care practices and procedures in handling of COVID-19 deceased bodies (suspected/confirmed) deposited in our mortuary located in a resource-poor setting as well as to explore their antemortem age/sex frequency distribution, sources and ascertain their deposition trends since the onset of the pandemic. We will compare our findings with existing literature to fill the identified knowledge gap in the death care field. To the best of our knowledge, this death care survey is the first of its kind in Nigeria, underscoring its profound importance in contributing to knowledge.

## Methods

**Study design**: this was a retrospective (January 2020 to September 2021) hospital-based cross-sectional survey at the mortuary unit of Department of Histopathology, University of Uyo Teaching Hospital (UUTH), Uyo, Akwa Ibom State, South-South region of Nigeria.

**Study setting**: UUTH is a 500-bed tertiary healthcare facility serving Uyo (a metropolitan city) and Akwa Ibom State. UUTH mortuary is a 500-body storage-capacity death care facility.

**Study participants**: all COVID-19 deceased bodies (suspected/confirmed) deposited in and cared for at UUTH mortuary within the study period. Note that most COVID-19 deceased bodies were initially received as suspected cases (and equally treated as positive cases) because of delays in confirmatory test completion, hence the inclusion of suspected and confirmed COVID-19 cases in this survey.

**Sample size**: all the COVID-19 deceased bodies brought in during the study period were included into this survey.

**Data collection**: elevant data were extracted from UUTH mortuary and COVID-19 isolation center registers. These included age, sex, hospital ward (death site), COVID-19 diagnostic status, death care practices and procedure type. Bias was minimized by crosschecking data from both locations with each other.

**Death care practices and procedures for deceased bodies of suspected COVID-19 cases in UUTH mortuary**: this protocol was adapted from ICRC and WHO recommendations, for death care of COVID-19 deceased bodies in our resource-poor setting. This protocol involved: 1) training of morticians on handling of COVID-19 deceased bodies (suspected/confirmed), including wear/use of Hazmat suit. 2) Designation of a mortuary room as a “COVID-19 embalming and body storage room”; 3) fabrication of the metallic embalming tank. 4) Modification of embalming fluid (Formal-Saline-Thymol concentrated solution); to ensure higher strength than routine embalming fluid, serves the purpose of preserving and preventing viral transmission through direct contact. 5) Procurement of bleach and sprayer for disinfection of deceased bodies and work surfaces/equipment/instruments.

**Procedure for reception of the deceased bodies of suspected COVID-19 cases at UUTH mortuary unit**: 1) The COVID-19 deceased bodies (suspected/confirmed) were received in single acrylic “zipped” body bags at the UUTH mortuary reception. These deceased bodies were deposited by healthcare personnel dressed in Hazmat suits (who disinfected the deceased bodies before bagging, and the body bags after bagging the deceased bodies). These personnel place the bagged deceased bodies on a designated metallic trolley bed. 2) The body bag is disinfected by our morticians (death care givers) dressed in Hazmat suits and thereafter wheel them to the “COVID-19 embalming and body storage room”. 3) The biodata and clinical summary form of the COVID-19 deceased bodies (suspected/confirmed) were received from other accompanying healthcare staff at our mortuary reception for documentation and identification purposes. We generated wrist band and ankle band identification tags for each deceased body.

**Nature and use of “COVID-19 embalming and body storage room”**: this room was designated for handling COVID-19 deceased bodies. It houses embalming tanks, concrete stones, zinc roofing sheets, metallic trolley beds, and waste baskets. The following were done here:

**For immersion embalmment**: 1) the body bag was laterally cut open on one side using a surgical blade creating a free anterior flap; taking care not to injure the deceased body within. This anterior flap was completely reflected to expose the dressed deceased body. 2) The deceased body was undressed using a pair of scissors to cut and remove the clothes. These clothes were placed in biohazard waste basket. 3) The wrist band and ankle band identification tags properly labeled with the deceased body´s name and mortuary number are applied to the right wrist and right ankle respectively. 4) The naked deceased body was transferred into the embalming tank and adequate volume of embalming fluid was then added gently to avoid splashing. The volume of embalming fluid used per time depends on the size of the deceased body(s). The aim of using this embalming tank was to completely submerge the deceased bodies. Hence, concrete stones were placed on the abdomen of floating deceased bodies to aid complete submersion. 5) The tanks were covered with zinc roofing sheets. These roofing sheets, held in place by concrete stones placed on top, prevent the escape of the embalming fluid. 6) The deceased´s clothes were transferred from the waste basket to a designated incineration site. At this site, the death care givers get disinfected and thereafter removed their Hazmat suits, which they burn along with the deceased´s clothes. 7) The submerged deceased body(s) are checked daily, by death care givers wearing Hazmat suits, for the first four days and thereafter it is left to embalm for one to two months. 8) Upon satisfactory embalmment, the deceased body(s) is removed, by death care givers wearing Hazmat suits, from the embalming tank and stored in body shelf. The deceased body is thereafter maintained using surface embalmment until the time it released for burial.

**For arterial embalmment**: 1) the steps 1, 2 and 3 of immersion embalmment were still applied here. In this procedure, routine protective mortuary clothings were used instead of Hazmat suits. 2) Embalming tank was also not used here, rather the naked deceased body was transferred to another metallic trolley bed and placed in a supine anatomical position and covered with modesty clothing sheet (Ankara fabric). Then, the upper anterior aspect of the right thigh is dissected with a surgical blade/dissecting forceps to locate the femoral artery (within the femoral triangle). 3) The femoral artery is cannulated with the tube of eight-liter-manual embalming pump. This embalming pump then injected embalming fluid into the deceased body through the femoral artery. Care is taken, while embalming, to touch the deceased body minimally. It takes 24 to 48 hours to fully embalm the deceased body. The deceased body is then transferred to the body shelf for storage and maintenance. 4) The waste management and body storage (post-embalmment) protocols remain the same here.

### Nature and use of the embalming tanks immersion embalmment.

**Material**: iron metal; locally fabricated and painted with black oil paint. 2) Tank dimensions and capacityis shown in Table 1. 3) Tank volumes: are shown in Table 1. 4) Metallic thickness: 0.2 cm

**Table 1:** aspect of the methodology, namely the dimensions, capacity and volume of the embalming tank as well as the composition of the embalming fluids used in the immersion and arterial embalming methods/procedures

Dimensions and capacity of embalming tanks		
Nature of tank	Dimensions of tank (cm)	Capacity of tank
Bigger tank	373 x 120 x 63	Houses two to four deceased bodies per time
Smaller tanks	371 x 60 x 35 each	Each house one deceased body per time
**Volumes of tanks**		
**Nature of tank**	**Volume (liters)**	**Maximum volume of embalming fluid usable**
Bigger tank	2,819.89	≈ 1,410 liters
Smaller tanks	779.1 each	≈ 390 liters
**Embalming fluid composition for immersion embalmment**		
**Chemical**		**Amount**
40% Formaldehyde		400 liters
Water		600 liters
Thymol crystals		5 grams/liter up to maximum of 20 grams/liter
Sodium Chloride (NaCl)		8.5 grams/liter
**Embalming fluid composition for arterial embalmment**		
**Chemical**		**Amount**
40% Formaldehyde		400 liters
Water		600 liters
Methylated spirit		40 liters
Glycerol		10 to 20 liters
Thymol crystals		5 grams/liter up to maximum of 20 grams/liter
Sodium Chloride (NaCl)		8.5 grams/liter

**Procedure for compounding embalming fluid**: embalming fluid composition for immersion embalmment: a) formal-saline-thymol concentrated solution: was compounded for a 1000 liters container as shown in Table 1. b) Embalming fluid composition for arterial embalmment: was compounded for a 1000 liters container as shown in Table 1. c) Nature of Personal protective equipment (PPE): 1) Hazmat suit for immersion embalmment; 2) Routine mortuary protective wear (namely: surgical face masks, plastic face shield, plastic eye goggles, latex hand gloves, acrylic/plastic aprons, surgical gowns, surgical caps, and rubber boots) for arterial embalmment.

**Procedure for COVID-19 diagnosis in Akwa Ibom State**: 1) specimen collection: nasopharyngeal and oropharyngeal specimen were collected with swab sticks from these suspected/confirmed COVID-19 patients antemortem. 2) Specimen transport to diagnostic laboratory: these nasopharyngeal and oropharyngeal swabs were transported in viral transport media (VTM) to the COVID-19 diagnostic laboratory of Infectious Disease Control Centre, Ituk Mbang, Akwa Ibom State, Nigeria. 3) Diagnostic test: COVID-19 positivity status was confirmed through SARS-CoV-2 reverse transcription polymerase chain reaction (RT-PCR) test at the COVID-19 diagnostic laboratory.

**Ethical considerations**: the research proposal for this survey was reviewed by the UUTH ethical committee and was approved and assigned the ethics approval number: UUTH/AD/S/96/VOL.XXI/630. This survey was subsequently done in accordance with “The Code of Ethics of the World Medical Association (Declaration of Helsinki)”. The identities of our participants were fully concealed (no personal identifier was used) in reporting. Furthermore, no harm was done to the participants or their living relatives.

**Data analysis**: all the data collected were entered into a research notebook and analyzed using Microsoft Excel 2016 edition. The results of this survey were presented as frequency tables and photographs.

## Results

**Figure 1:**
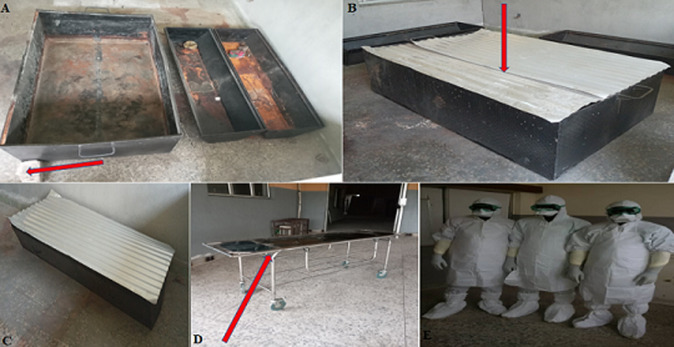
(A - E) showing equipment used for “Immersion embalmment with embalming tank and Hazmat suit use”: (A) shows the “bigger” and “smaller” embalming tanks side by side; Also shown is one of the concrete stones (red arrow) used in the embalming procedure; (B) shows two unfolded zinc roofing sheets [red arrow] used in covering the “bigger” embalming tanks during the embalming procedure; (C) shows an unfolded zinc roofing sheet used in covering the “smaller” embalming tanks during the embalming procedure; (D) shows a metallic trolley bed (red arrow) used in conveying deposited bodies of suspected and confirmed COVID-19 cases in UUTH mortuary from the point of reception of the deceased bodies to the COVID-19 embalming room; (E) shows three death care givers (morticians) fully kitted in Hazmat suits used in handling deposited bodies of suspected and confirmed COVID-19 cases in UUTH mortuary

**Figure 2:**
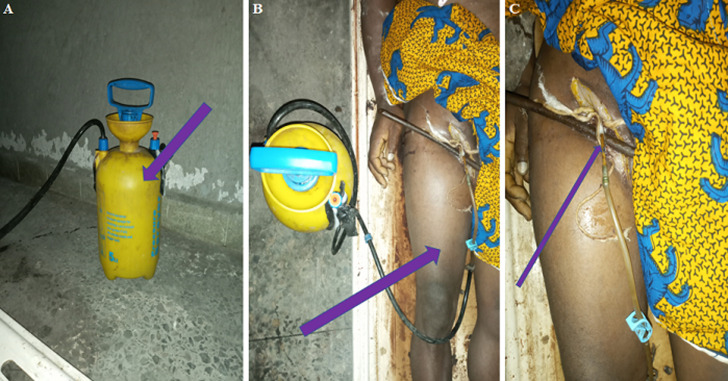
photographs (A,B,C) showing “arterial embalmment with embalming pump”: (A) shows a manual 8-liter embalming pump (purple arrow); (B) shows the connection of the manual embalming pump to the right femoral artery in the right thigh of deceased body (purple arrow); (C) shows a closer look of the tube of the manual embalming pump to the right femoral artery (purple arrow); note that death care givers wearing routine mortuary protective wear were not shown in these photographs

In this study we found 28 deposited deceased bodies of COVID-19 cases (22 positives, five negatives and one probable) from January 2020 to September 2021. Notably, there were 14 deposited deceased bodies of COVID-19 cases (10 positives, three negatives and one probable) from January to December 2020, accounting for 4.69% of depositions in UUTH mortuary within this period (14/298) and 14 deposited deceased bodies of COVID-19 cases (12 positives and two negatives) from January to September 2021, accounting for 8.05% of depositions in UUTH mortuary within this period (14/174). We found that most of these deposited COVID-19 deceased bodies (suspected/confirmed) were within the 6^th^ and 7^th^ decades of life (25.93% each). The least age, highest age, mean, median, and mode of the ages were 25, 81, 57.78 ± 14.57, 60 and 60 years respectively (Table 2). Furthermore, most of them were males (71.43%), giving a male to female ratio (M/F) of 2.5/1 (Table 2). We also found that most (64.29%) of these deceased bodies were managed at University of Uyo Teaching Hospital (UUTH) COVID-19 Isolation Center (Table 3). Interestingly, we found that these 28 deposited COVID-19 deceased bodies (suspected/confirmed) accounted for 5.93% of depositions in UUTH mortuary within this period (28/472), (Table 3). Importantly, we found that the most common type of death care provided to these deceased bodies was “Immersion embalmment with embalming tank” by Hazmat suit kitted death care givers (75%). The “bigger” embalming tank can harbor two to four deceased bodies per session of embalmment by immersion while the two “smaller” embalming tanks can only harbor one deceased body per session of embalmment by immersion (Table 3 and Figure 1). Importantly too, we ascertained that the peak periods of deposition of these deceased bodies were April, June and July 2020, and February and July 2021 (Table 4). Notably too, we found that the type of death care given since June 2021 has been “Arterial embalmment” with embalming pump by routine mortuary protective wear kitted death care givers (Table 4 and Figure 2).

**Table 2:** frequency distribution of the age and sex of study participants

Frequency distribution of the age ranges of the deposited COVID-19 deceased bodies (suspected and confirmed) in UUTH mortuary within the study period		
Age ranges	Frequency	Percentage
0-10	0	0
11-20	0	0
21-30	2	7.41
31-40	1	3.70
41- 50	6	22.22
51-60	7	**25.93**
61-70	7	**25.93**
71-80	3	11.11
81-90	1	3.70
1-100	0	0
**Total**	**27**	**100**
**Frequency distribution of the sexes of the deposited COVID-19 deceased bodies (suspected and confirmed) in UUTH mortuary within the study period**		
**Sex**	**Frequency**	**Percentage**
Male	20	71.43
Female	8	28.57
**Total**	**28**	**100**

**Table 3:** frequency distribution of the origins of the COVID-19 deceased bodies, their diagnostic status and the type of death care practice and procedure they received

Frequency distribution of the sources of the deposited COVID-19 deceased bodies (suspected and confirmed) in UUTH mortuary within the study period		
Ward	Frequency	Percentage
Brought-in-dead (BID)	4	14.29
UUTH COVID-19 Isolation Center	18	64.29
Ituk Mbang Infectious Disease Control Center	3	10.71
Ibom Multispecialist Hospital COVID-19 Isolation Center	1	3.57
UUTH Ward-not-specified	2	7.14
**Total**	**28**	**100**
**Frequency distribution of the COVID-19 diagnosis status of the deposited COVID-19 deceased bodies (suspected and confirmed) in UUTH mortuary within the study period**		
**COVID-19 Status**	**Frequency**	**Percentage**
Confirmed positive	22	78.57
Confirmed negative	5	17.86
Probable	1	3.57
**Total**	**28**	**100**
**Frequency distribution of the type of death care practice and procedureadministered to the deposited COVID-19 deceased bodies (suspected and confirmed) in UUTH mortuary within the study period**		
**Type of death care practice and procedure**	**Frequency**	**Percentage**
Immersion embalmment with embalming tank and Hazmat suit use	21	75
Arterial embalmment with manual embalming pump and routine mortuary protective wear use	7	25
**Total**	**28**	**100**

**Table 4:** frequency distribution table showing the relationship between the respective months of the study period and the type of death care practice and procedure administered to deposited COVID-19 deceased bodies (suspected and confirmed) in UUTH mortuary

Month/Year of Deposition	Type of Death Care Practice and Procedure	Frequency	Percentage
Jan-20	0	0	0
Feb-20	0	0	0
Mar-20	0	0	0
Apr-20	Immersion embalmment with embalming tank and Hazmat suit use	3	10.71
May-20	Immersion embalmment with embalming tank and Hazmat suit use	2	7.14
Jun-20	Immersion embalmment with embalming tank and Hazmat suit use	3	10.71
Jul-20	Immersion embalmment with embalming tank and Hazmat suit use	3	10.71
Aug-20	Immersion embalmment with embalming tank and Hazmat suit use	2	7.14
Sep-20	0	0	0
Oct-20	Immersion embalmment with embalming tank and Hazmat suit use	1	3.57
Nov-20	0	0	0
Dec-20	0	0	0
Jan-21	Immersion embalmment with embalming tank and Hazmat suit use	2	7.14
Feb-21	Immersion embalmment with embalming tank and Hazmat suit use	4	14.29
Mar-21	Immersion embalmment with embalming tank and Hazmat suit use	1	3.57
Apr-21	0	0	0
May-21	0	0	0
Jun-21	Arterial embalmment with embalming pump and routine mortuary protective wear	1	3.57
Jul-21	Arterial embalmment with embalming pump and routine mortuary protective wear	4	14.29
Aug-21	Arterial embalmment with embalming pump and routine mortuary protective wear	1	3.57
ep-21	Arterial embalmment with embalming pump and routine mortuary protective wear	1	3.57
**Total**		**28**	**100**

## Discussion

In this study, we aimed to survey the death care practices and procedures in handling the deceased bodies of COVID-19 cases (suspected/confirmed) in UUTH mortuary thus far since the beginning of the COVID-19 pandemic. This survey also aimed to know the age/sex distribution of these deceased bodies, the trends in their death care and the nature thereof, to appraise the qualitative effectiveness of our death care practices in preserving these deceased bodies as well as in reducing the risk of transmission of SARS-CoV-2 to death care workers (body handlers). All our study participants were embalmed, with about three-quarters (75%) of them being embalmed by immersion. Amongst these deceased bodies, we found that about a quarter (25.93% each) of them were within the sixth and seventh decades of life, with about three-quarters (71.43%) of them being males, and about three-quarters (64.29%) of them came from UUTH COVID-19 Isolation Center. Notably, COVID-19 deceased bodies constituted about one-sixteenth (5.93%) of deceased bodies deposited in UUTH mortuary. The peak periods of their deposition were within the first halves of these two years of COVID-19 pandemic.

As regards the death care (preservation/storage) provided to COVID-19 deceased bodies, it is important to note that our preferred method of preservation (for temporary storage before burial or cremation) for all the deceased bodies was embalmment (specifically immersion and arterial embalmment methods). The rationale for this preference is that formaldehyde-based embalming fluids are excellent chemicals for preservation of deceased bodies (whether deceased bodies associated with natural death/non-infectious diseases or associated with infectious diseases like SARS-CoV-2 infection) as well as for prevention of transmission of the SARS-CoV-2, through direct contact, aerosols and droplets generation, to death care workers [4,7,15,18,31-33]. We initially used “Immersion embalmment with embalming tank and Hazmat suit use” as the preferred embalmment method, providing for three-quarters (75%) of these deceased bodies but have now switched over to “Arterial embalmment with embalming pump and routine mortuary protective wear use”. We worked with WHO/ICRC recommendation of minimal body manipulation as well as application of general infection prevention/control precautions during the embalming process. Furthermore, during arterial embalmment procedures we avoided creating extra vascular outlet channels for bloodletting. Also, we did not practice cavity embalmment, thus minimizing body manipulation and avoiding splashing with attendant aerosol/droplets generation from the deceased body. This method was consistent with the study by Ezugworie and Anibeze [32].

Notably, this switch from immersion to arterial embalmment was primarily borne of the non-sustainability of continued use of enormous amounts of embalming fluid for immersion embalmment, which was quite expensive to maintain in our resource-poor environment. Furthermore, this switch was consistent with studies by Alishaq and Jeremijenko, Late SV *et al*. Swain R *et al*. and O´Keeffe, who found that death care givers rarely contracted SARS-CoV-2 at their work places but rather in their communities, showing the effectiveness of disinfection of their environmental/work surfaces [3,4,22,30]. Similarly, this switch was also consistent with the COVID-19 deceased body handling guidelines by RCPath, Public Health England, WHO/ICRC/IFRC, etc. who advocated exemption of immunocompromised or > 60-year-old death care workers, proper hand hygiene, reduced manipulation of the deceased bodies, effective disinfection (with hospital grade bleach [1% Sodium Hypochlorite] or 70% ethanol) of environmental/work surfaces, and correct use of standard personal protective equipment (PPE) [3-7,15-19,21,22,25,30-35]. We applied all these recommendations while making this switch and still had satisfactory deceased bodies preservation with protection of our death care workers. By implication, COVID-19 deceased bodies can be well cared for in a resource poor like ours or elsewhere by applying these same principles of death care.

Interestingly, we did not refrigerate any of these deceased bodies because as a department we reached a consensus that our mortuary 24-chamber body freezer was not ideal in reducing the risk of SARS-CoV-2 transmission from COVID-19 deceased bodies during their death care. This consensus was founded on the understanding that SARS-CoV-2 can persist on environmental/work surfaces and deceased bodies (body fluids and tissue) for up to nine days, particularly if death occurred within 14 days of COVID-19 diagnosis in symptomatic cases [4,6,7,15,23]. However, this finding was inconsistent with the studies by Late SV *et al*., Fineschi V *et al*., Swain R *et al*. as well as in the WHO/ICRC/IFRC and NCDC COVID-19 guidance documents for handling deceased COVID-19 bodies (suspected/confirmed) who strictly recommended storage in body freezers as the preferred method of death care for COVID-19 deceased bodies [3,17,21-23,27,29]. The rationale for their preference was that there is reduced manipulation of deceased bodies during cold storage, as against embalming, thus reducing the risk of transmission of SARS-CoV-2, through aerosols and droplets generation, to death care workers to the barest minimum. This recommendation was the basis of our minimal deceased body manipulation even while embalming.

As regards the age/sex distribution of COVID-19 deceased bodies, it is important to note that about a quarter (25.93%) of these deceased bodies were within the sixth and seventh decades of life. These findings show that most of these deceased bodies were individuals within the middle age to early elderly age; altogether comprising about half (51.86%) of all the COVID-19 deceased bodies (suspected/confirmed). These findings agree with the study by O´Driscoll M *et al*. who found that individuals aged 65 years and above were most likely to die from COVID-19 [12]. Furthermore, the ages of these deceased bodies ranged from 25-81 years, while their mean and median ages were 57.78 and 60 years respectively. This finding is inconsistent with another COVID-19 study in our environment, though in living persons, by Udoette SB *et al*. who found the age range of 11- 63 years and mean age of 41.5 years [11]. This difference in age ranges could be because of our study´s longer duration, while the difference in mean age could be because their study involved a larger sample size. Also, our study focused solely on deceased individuals who were majorly (51.86%) in the sixth to seventh decades of life. Similarly, in another Nigerian COVID-19 study, also in living persons, by Osibogun A *et al*. the median age was 43 years [10]. This difference in median age could also be because of their larger sample size and that our study focused solely on deceased individuals, majorly (51.86%) in their sixth to seventh decades of life. Importantly too, about three-quarters (71.43%) of these deceased bodies were males, with a male to female ratio (M/F) of 2.5/1. These findings show that males were three times more likely to die from COVID-19 than females in our environment. These findings agree with the studies by Osibogun A *et al*. and Udoette SB *et al*. who found 65.8% and 70.1%, respectively, of males having COVID-19 [10,11]. This shows that these males, predominantly sick with COVID-19, were more likely to die from it. The immediate clinical implication of this is that there needs to be more focus on preventing the co-morbidities (associated with COVID-19 mortality) especially in males particularly those aged 51 to 70 years in our environment.

Notably, as regards the sources of the COVID-19 deceased bodies, about three-quarters (64.29%) of these deceased bodies were managed at the University of Uyo Teaching Hospital (UUTH) COVID-19 Isolation Center. This finding shows that the bulk our survey participants died at UUTH COVID-19 Isolation Center. It is of note, however, that there were 42 deaths in individuals with confirmed COVID-19 in Akwa Ibom State within our study period, hence only 22/42 (52.38%) of these deaths were cared for in UUTH mortuary [9]. Regrettably, data on death care for the rest (47.62%) of these deceased bodies is not available; this calls for further study. Interestingly, the deceased bodies of COVID-19 cases (suspected/confirmed) constituted about one-sixteenth (28/472; 5.93%) of depositions in UUTH mortuary within our study period. This finding shows that our 500-body storage capacity mortuary was not overwhelmed for death care space. However, several COVID-19 guidelines on handling deceased bodies recommend building larger facilities in preparedness for effective mass casualty death care for COVID-19 associated deceased bodies in case the mortality rate of this pandemic escalates [5-7,15-18,21,26,29,34]. By implication, we need to expand our mortuary facility to be prepared for potential escalations. As regards the trends in deposition, it was interesting to note that the peak periods of these deceased bodies deposition were April, June, and July for 2020, and February and July for 2021. These findings show that COVID-19 death rate maybe higher in the first halves of the two years affected by the COVID-19 pandemic (that is if deceased bodies deposition rate in our mortuary equates COVID-19 death rate in our environment). Notably, July 2021 peak period of COVID-19 deceased bodies deposition coincides with SARS-CoV-2 delta variant surge in Akwa Ibom State [9].

Finally, the next research step to follow up this study will be to conduct a robust, possibly national, multicenter survey of death care practices and procedures in handling COVID-19 deceased bodies in mortuaries of tertiary healthcare facilities in the six geo-political zones of Nigeria. This will aid filling the knowledge gap in death care practices and procedure in our environment. Secondly, to conduct a multicenter questionnaire-based survey of the knowledge-base and competence of death care workers across Nigeria, to identify the key areas in need of continuous improvement for excellence in Total Quality Management Systems (QMS) in the death care field. Thirdly, to conduct a multicenter virological/molecular biological study in COVID-19 Isolation Centers and tertiary healthcare facilities´ mortuaries, to determine the presence, niches/sites, mechanism of action and persistence/duration of viability of SARS-CoV-2 on/in their associated deceased bodies as well as places/instruments/equipment of death care, across Nigeria. This will help develop accurate/effective infection control and prevention precautions/measures/protocols in handling COVID-19 deceased bodies.

**Limitations**: the limitations of this study were derived from its retrospective nature. Thus, we had no direct control of our study variables. Secondly, given our small sample size, our findings cannot be accurately extrapolated to the general population. Thirdly, our study was done in one location, thus our findings cannot be accurately extrapolated to the general population. Fourthly, our mortuary´s deceased body release practices/procedures and the effect of COVID-19 pandemic on burial practices were not surveyed. Sixthly, the death care practices/procedures in handling COVID-19 deceased bodies in private-owned mortuaries across Uyo and Akwa Ibom State were not surveyed.

## Conclusion

In conclusion, our retrospective survey of COVID-19 deceased bodies death care practices/procedures in UUTH mortuary found, we found that all were embalmed, with 75% being embalmed by immersion. The peak periods of their depositions were within the first halves of the two years affected by the COVID-19 pandemic. Notably, 51.86% of them were within the 6^th^ and 7^th^ decades of life, 71.43% were males, 64.29% arrived from UUTH COVID-19 Isolation Center, and they constituted 5.93% of depositions. Thus, we provided death care (particularly through embalmment) for COVID-19 deceased bodies in our resource poor setting using locally derived innovative means while adhering to infection prevention/control measures to protect our death care workers.

### What is known about this topic

- *Numerous management (death care) guidance documents;*
- *But very few research-based information on death care of deceased bodies of individuals who died from COVID-19*.

### What this study adds

- *Death care workers, particularly those practicing in resource poor settings, like ours, can provide death care (majorly through embalmment) for COVID-19 deceased bodies, suspected/confirmed, using locally derived innovative means;*
- *While still adhering to infection prevention and control measures to reduce to the barest minimum the risk of transmission of SARS-CoV-2 in the mortuary*.
